# Mycorrhizal inoculation success depends on soil health and crop productivity

**DOI:** 10.1093/femsle/fnaf031

**Published:** 2025-03-12

**Authors:** Ido Rog, Marcel G A van der Heijden, Franz Bender, Raphaël Boussageon, Antonin Lambach, Klaus Schlaeppi, Natacha Bodenhausen, Stefanie Lutz

**Affiliations:** Plant–Soil Interactions, Agroecology and Environment, Agroscope, Reckenholzstrasse 191, 8046 Zurich, Switzerland; Department of Plant and Microbial Biology, University of Zurich, Zollikerstrasse 107, 8008 Zurich, Switzerland; Plant–Soil Interactions, Agroecology and Environment, Agroscope, Reckenholzstrasse 191, 8046 Zurich, Switzerland; Department of Plant and Microbial Biology, University of Zurich, Zollikerstrasse 107, 8008 Zurich, Switzerland; Plant–Soil Interactions, Agroecology and Environment, Agroscope, Reckenholzstrasse 191, 8046 Zurich, Switzerland; Department of Plant and Microbial Biology, University of Zurich, Zollikerstrasse 107, 8008 Zurich, Switzerland; Plant–Soil Interactions, Agroecology and Environment, Agroscope, Reckenholzstrasse 191, 8046 Zurich, Switzerland; Department of Plant and Microbial Biology, University of Zurich, Zollikerstrasse 107, 8008 Zurich, Switzerland; Plant–Soil Interactions, Agroecology and Environment, Agroscope, Reckenholzstrasse 191, 8046 Zurich, Switzerland; Department of Plant and Microbial Biology, University of Zurich, Zollikerstrasse 107, 8008 Zurich, Switzerland; Plant Microbe Interactions, Department of Environmental Sciences, University of Basel, 4056 Basel, Switzerland; Department of Soil Sciences, Research Institute of Organic Agriculture (FiBL), 5070 Frick, Switzerland; Plant–Soil Interactions, Agroecology and Environment, Agroscope, Reckenholzstrasse 191, 8046 Zurich, Switzerland; Department of Plant and Microbial Biology, University of Zurich, Zollikerstrasse 107, 8008 Zurich, Switzerland

**Keywords:** arbuscular mycorrhizal fungi, soil health, field inoculations, crop productivity, sustainable agriculture, mycorrhizal growth response

## Abstract

As the human population grows, so does the demand for higher agricultural yields. As a result, agricultural intensification practices are increasing while soil health is often declining. Integrating the benefits of microorganisms into agricultural management systems can reduce the need for external resource inputs. One particular group of plant symbionts that can help plants to acquire additional nutrients and promote plant growth are arbuscular mycorrhizal fungi (AMF). The application of AMF in agricultural practice has been hampered by the variability in the success of mycorrhizal inoculation and the lack of consistency in different fields. Here, we tested whether it is possible to predict mycorrhizal inoculation success based on soil health and productivity. We hypothesized higher inoculation success on fields with poor soil health because in such fields, mycorrhiza can improve nutrient uptake and biotic resistance to pathogens. We calculated a soil health index by aggregating six biotic and abiotic variables from 54 maize fields and tested its correlation with the mycorrhizal growth response (MGR). The MGR was linked to soil health and significantly higher in less healthy soils and less productive fields. This implies that soil inoculation with AMF has most potential in fields with poor soil health and low productivity. Based on these findings, we propose a soil health framework that highlights the potential benefits of AMF field inoculation.

## Teaser

We show that arbuscular mycorrhizal fungi benefit crops most in less healthy soils and less productive fields, providing a framework for improving AMF application and supporting global sustainability goals.

Agricultural intensification aims to meet the growing food demand by increasing productivity on existing land applying high doses of agrochemicals. However, this approach has resulted in high environmental costs, particularly in terms of reduced soil health (Lehmann et al. 2020). As a consequence, there is much interest to restore natural processes and integrate beneficial soil microbes, such as arbuscular mycorrhizal fungi (AMF). AMF are a well-known group of plant symbionts that form mutualistic associations with two-thirds of all land plants, including most crops. With their dense hyphal networks, AMF can enhance nutrient and water uptake beyond the root depletion zone, and significantly increase the plant phosphorus and nitrogen uptake (Hodge et al. 2010, Verbruggen et al. 2012, Martin and van der Heijden 2024). In addition to improving abiotic aspects of soil health (e.g. nutrient bioavailability and soil structure), recent findings have also linked AMF to the biotic soil health perspective by improving yield in soils with high pathogen abundance (Lutz et al. 2023). Both the abiotic and biotic AMF functions can help to reduce dependence on external inputs and support sustainable agriculture (Bender et al. 2016, Schütz et al. 2018).

Intensively fertilized and managed agricultural soils often reduce the abundance and diversity of AMF (Verbruggen et al. 2012, Peng et al. 2024), compared to systems such as grasslands, where AMF are strongly linked to primary productivity (Romero et al. 2024). Therefore, field inoculations of AMF in agricultural soils have a high potential to provide or restore the associated functions of AMF with crops, and to enrich depleted native AMF pools. However, outcomes for crop performance are inconsistent due to the lack of knowledge about the factors that contribute to crop response to AMF inoculation, such as AMF genotypes (Angelard et al. 2014), crop genotypes, and compatibility (Sawers et al. 2008, Thirkell et al. 2022), as well as other biotic and abiotic factors (Berger and Gutjahr 2021). Another factor contributing to the inconsistency and variability of AMF response in the field is the quality of the AMF propagules present on the market, which does not lead to a functional symbiosis and damages the reputation of these new products (Salomon et al. 2022, Koziol et al. 2024).

In a recent study, we observed that AMF inoculation can increase crop yield in Swiss arable fields, with an average increase of 6% across all 54 fields (Lutz et al. 2023), suggesting AMF as an encouraging biological supplement. However, the mycorrhizal growth response (MGR) varied highly from +40% to −12%. Soil microbiome indicators (mainly pathogenic soil fungi such as *Trichosporon* sp., *Myrothecium* sp., and *Olpidium* sp.) and soil physico–chemical properties (e.g. magnesium, microbial biomass carbon, and manganese) could predict 86% of the variation in AMF inoculation success. However, a comprehensive understanding of the specific conditions that maximize the benefits of AMF in the field remains incomplete.

**Figure 1.**
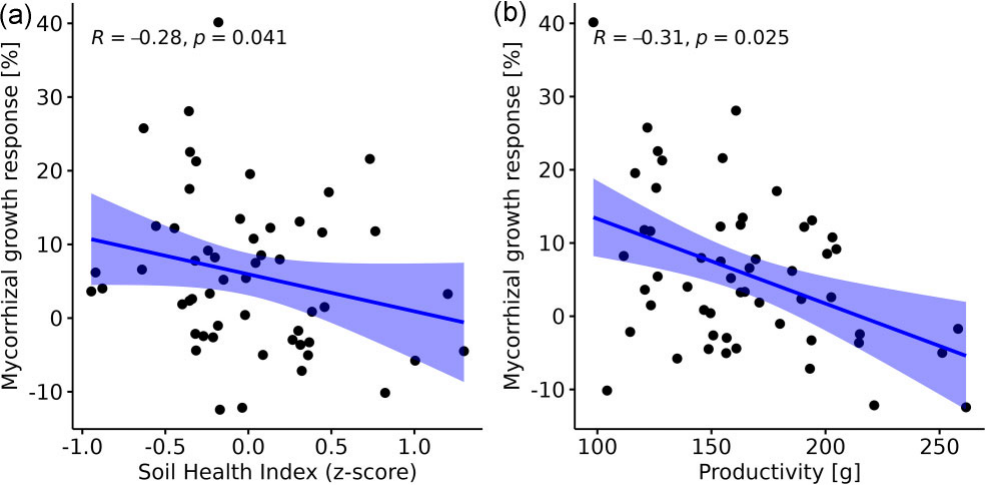
High MGR in fields with less healthy soils and low fertility. (a) Negative significant Pearson correlation between MGR and soil health index for arable land. (b) Negative and significant Pearson correlation between MGR and plant biomass (noninoculated plants). Data are based on 54 inoculated plots over 3 years in Switzerland, for more information see Lutz et al. (2023). Soil health was calculated according to Romero et al. (2024) and MGR according to Köhl et al. (2016). Soil health is calculated on the basis of six explanatory variables (microbial biomass carbon, soil AMF richness, inverse pathogen abundance, plant-available phosphorus, organic carbon, and plant-available mineralized nitrogen) equally weighted using Z-score transformation.

Here, we used the yield data and soil health properties from 54 maize fields and tested whether the benefits of mycorrhizal fungal inoculation were related to agricultural soil health and productivity (crop yield without AMF inoculation). We specifically hypothesized that the benefits of AMF inoculation would be greatest in low productivity soils with poor health, where mycorrhiza can improve nutrient uptake and biotic resistance to pathogens. The soil health index for arable land was calculated based on an aggregate of three biotic and three abiotic factors: microbial biomass carbon, soil AMF richness, inverse pathogen abundance, plant-available phosphorus, organic carbon, and plant-available mineralized nitrogen. We followed the approach described in Romero et al. (2024) with slight modifications due to data availability (i.e. richness of N-fixing bacteria was replaced by mineralized nitrogen to include plant-available nitrogen and the water infiltration potential was not included). We found a significant negative correlation between MGR and soil health (*R* = −0.28, *P* = .041) (Fig. 1a). In addition, when calculating the potential of AMF inoculation in relation to plant productivity, we also found a significant negative correlation (*R* = −0.31, *P* = .025) (Fig. 1b). These results highlight the potential of AMF inoculation to maximize yield potential in less healthy soils with low productivity. Moreover, the results show the limitations of AMF inoculation as a tool to increase yield in healthy soils where plant size is already maximized (Fig. 1). Under these conditions, AMF may also induce unintended adverse growth effects. Further research is needed to understand the mechanisms underlying the occasional adverse effects of AMF in healthy soils to ensure optimized application strategies for sustainable agriculture.

**Figure 2.**
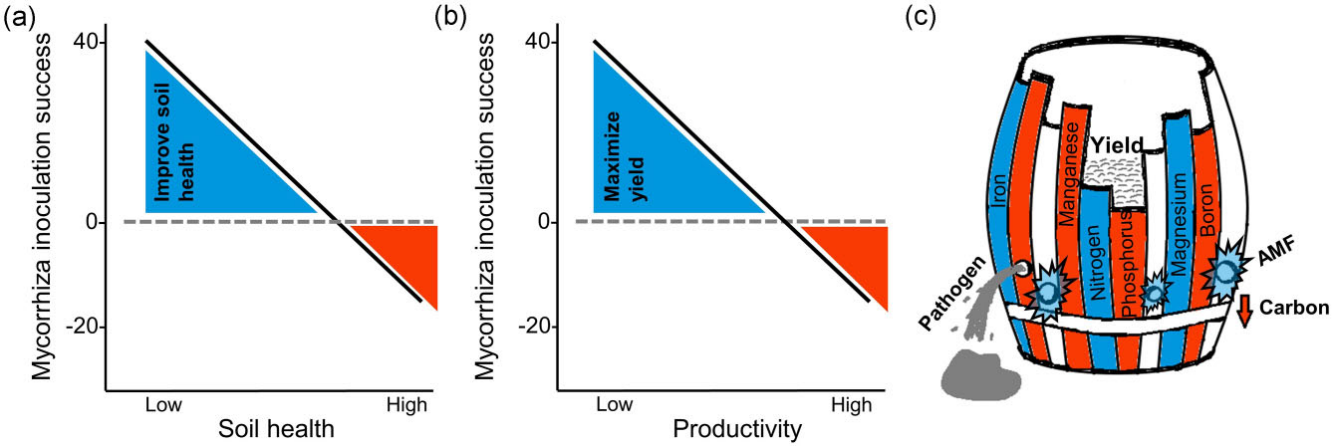
Schematic illustration of AMF inoculation potential to maximize sustainable yield. AMF inoculation maximizes yield in less healthy soils. High mycorrhizal inoculation success is observed in fields with low soil health (a) and with low soil productivity (b). Negative mycorrhizal inoculation success can occur in very healthy soils with high plant productivity. (c) An adapted version of Liebig’s law of the minimum illustrates how AMF inoculation can increase yield. In this model, elements with a positive effect on MGR are represented in blue, while those with a negative effect on MGR are shown in red (Lutz et al. 2023). Soil pathogens are depicted as “holes,” with AMF acting as “hole covers,” blocking pathogen access and yield loss. The metal ring of the barrel, which stabilizes the whole barrel, represents soil carbon with a negative effect on MGR.

Although AMF inoculations can enhance yields even in intensively managed fields with fertilizer and pesticide inputs (see Lutz et al. 2023), their potential is even greater in less healthy, low-productivity soils. Here, we introduce a soil health framework for optimizing AMF inoculation to maximize crop yields in resource-limited fields (Fig. 2). AMF inoculation has the potential to significantly contribute to sustainable agriculture by reducing the need for chemical fertilizers and pesticides, improving food security, and addressing critical Sustainable Development Goals (SDGs), including SDG 2 (Zero Hunger) and SDG 3 (Good health and well-being). The technology behind AMF-based biofertilizers and biocontrol products has the potential to increase plant nutrient uptake, improve drought resilience, and reducing fertilizer requirements (Augé et al. 2001, Cavagnaro et al. 2015). For example, AMF can increase plant drought tolerance by expanding the root system water uptake zone and plant–water relations (Kakouridis et al. 2022), which is critical for sustainable water management and addressing SDG 6 (clean water and sanitation). In addition to improving soil health, AMF enhance plant resilience to various environmental stresses, including pathogen attack. AMF colonize plant roots, creating a physical barrier that limits pathogen access, while stimulating the plant’s immune response and suppressing soil-borne diseases (Branco et al. 2022). This mutualistic relationship highlights the role of AMF in sustainable agriculture as a natural defense against pathogens, contributing to SDG 3 (Good Health and Well-being).

Agricultural intensification can reduce the nutrient uptake capacity of native AMF, thereby reducing their ability to promote the plant’s nutrient use efficiency (Riedo et al. 2021, Edlinger et al. 2022). Enriching the rhizosphere with beneficial AMF while using less intensive management systems, can improve soil fertility and potentially increase crop yield (Fig. 2). According to Liebig’s law, plant growth is limited by the smallest amount of nutrient supplied relative to the plant’s needs (Liebig 1840). To increase crop yield, it is essential to address the availability of the limiting element, while changing the availability of the other elements will have no effect. Phosphorus, nitrogen, and potassium are often the limiting elements in agricultural soils, which is why they are commonly supplemented through fertilizer. Some soil nutrients can be taken up by the plant root, but many are bound to the soil minerals and become insoluble and less available (Hart et al. 2004). Microbes, particularly AMF in association with hyphosphere bacteria, can release soil-bound nutrients to enhance plant nutrient uptake and growth (Anckaert et al. 2024, Zhang et al. 2024). AMF, in general, and specifically *Rhizoglomus irregularis*, which is commonly used as an inoculum, have been shown to increase the phosphorus uptake of inoculated plants and decrease the nitrogen-to-phosphorus ratio (Boussageon et al. 2022, Tessier and Raynal 2003, Joner et al. 2000). Consistently, soil parameters predicted ~29% of the MGR of maize in the field (Lutz et al. 2023). Among the macroelements, nitrogen and magnesium abundance had a positive effect, while phosphorus abundance had a negative impact on MGR. Other microelements such as manganese and boron had a negative effect, whereas iron contributed positively.

Mycorrhizae have been described as “nature’s response to the law of the minimum supply” because of their ability to improve plant access to limiting nutrients in the soil (Read 1991, Johnson et al. 2015). Here, we propose that in fields with low soil health mycorrhizal inoculation can improve the availability of essential elements to the plant and increase plant yield to its maximum potential. Liebig’s law and the “barrel” analogy illustrate how abiotic factors of agricultural soil health can limit crop yield (Fig. 2c). Similarly, biotic factors of soil health and pathogen abundance can also limit crop yield (Singh et al. 2023). In addition to improving nutrient availability in natural ecosystems, recent findings show that high pathogen abundance in agricultural systems is the main predictor of high MGR (Lutz et al. 2023). Thus, to the well-known “barrel” analogy of Liebig’s law, we have now added soil pathogens as “holes,” with AMF acting as “hole covers,” blocking pathogen access and yield loss (Fig. 2c). However, the detailed mechanisms underlying AMF–pathogen interactions remain unknown. Another critical variable in the biotic factor of soil health is microbial carbon, which can contribute to sustainable yield (Ngatia et al. 2021, Toda et al. 2023), and which we depict as the metal ring of the barrel that stabilizes the whole barrel. Our new soil health framework adds the biotic factors to the well-known Liebig’s law of the minimum and highlights the potential of AMF field inoculation to enhance sustainable yield.

In conclusion, we propose that targeted AMF inoculation can improve plant nutrient availability and crop growth, particularly in fields with low soil health and high pathogen burden. At present, our results are based on a crop known to be rather mycotrophic, and we only have data on a single maize genotype and AMF strain; further experiments involving other crop genotypes and AMF consortia are needed to broaden the potential. Moreover, the results presented here are based on general patterns and links between variables (e.g. MGR and soil health) comparing 54 fields. Experimental studies are needed to verify our findings (e.g. manipulate soil health on a specific experimental field with the same climate and abiotic soil characteristics, and inoculate plots with low and high soil health). The proposed framework of the biotic and abiotic benefits of AMF inoculation can assist scientists, farmers, and policy makers in using AMF as a biofertilizer and biocontrol product to improve sustainable yields. By considering soil health for arable land and specifically pathogen abundance, our framework supports the use of AMF to improve yield potential, thereby strengthening sustainable agricultural practices in line with the global SDGs.
